# Extracellular vesicles as potential biomarkers for diagnosis and recurrence detection of hepatocellular carcinoma

**DOI:** 10.1038/s41598-024-55888-8

**Published:** 2024-03-04

**Authors:** Mazen A. Juratli, Nicola S. Pollmann, Elsie Oppermann, Annika Mohr, Dhruvajyoti Roy, Andreas Schnitzbauer, Sabine Michalik, Thomas Vogl, Nikolas H. Stoecklein, Philipp Houben, Shadi Katou, Felix Becker, Jens Peter Hoelzen, Andreas Andreou, Andreas Pascher, Wolf O. Bechstein, Benjamin Struecker

**Affiliations:** 1 Department of General, Visceral and Transplant Surgery, Muenster University Hospital, Muenster University, Muenster, Germany; 2 Department of General, Transplant and Thorax Surgery, Frankfurt University Hospital, Goethe University, VisceralFrankfurt, Germany; 3 Department of Breast Surgical Oncology, The University of Texas MD Anderson Cancer Center, Houston, TX USA; 4 Department of Diagnostic and Interventional Radiology, Frankfurt University Hospital, Goethe University, Frankfurt, Germany; 5 General, Visceral and Pediatric Surgery, University Hospital and Medical Faculty of the Heinrich-Heine-University Düsseldorf, Düsseldorf, Germany

**Keywords:** Hepatocellular carcinoma, Non-malignant liver disease, Extracellular vesicles, Surgery, Recurrence, Cancer, Biomarkers, Gastroenterology

## Abstract

Hepatocellular carcinoma (HCC) is the most common primary malignant liver tumor and a leading cause of cancer-related deaths worldwide. However, current diagnostic tools are often invasive and technically limited. In the last decade, non-invasive liquid biopsies have transformed the field of clinical oncology, showcasing the potential of various liquid-biopsy derived analytes, including extracellular vesicles (EVs), to diagnose and monitor HCC progression and metastatic spreading, serving as promising novel biomarkers. A prospective single-center cohort study including 37 HCC patients and 20 patients with non-malignant liver disease (NMLD), as a control group, was conducted. Serum EVs of both groups were analyzed before and after liver surgery. The study utilized microbead-based magnetic particle sorting and flow cytometry to detect 37 characteristic surface proteins of EVs. Furthermore, HCC patients who experienced tumor recurrence (R-HCC) within 12 months after surgery were compared to HCC patients without recurrence (NR-HCC). EVs of R-HCC patients (n = 12/20) showed significantly lower levels of CD31 compared to EVs of NR-HCC patients (*p* = 0.0033). EVs of NMLD-group showed significantly higher expressions of CD41b than EVs of HCC group (*p* = 0.0286). The study determined significant short-term changes in CD19 dynamics in EVs of the NMLD-group, with preoperative values being significantly higher than postoperative values (*p* = 0.0065). This finding of our pilot study suggests EVs could play a role as potential targets for the development of diagnostic and therapeutic approaches for the early and non-invasive detection of HCC recurrence. Further, more in-depth analysis of the specific EV markers are needed to corroborate their potential role as diagnostic and therapeutic targets for HCC.

## Introduction

Hepatocellular carcinoma (HCC) is the most common primary liver cancer, with an increasing incidence and high recurrence rate, which negatively impacts prognosis and overall survival^1^. Currently, hepatic resection, radiofrequency ablation, and liver transplantation represent curative therapeutic options for HCC at early-stages. Recurrence rates of early-stage HCC remain high by 35% within the first year^2^ and resemble a crucial prognostic factor since early recurrence is associated with developing incurable disease^3,4^. The liver represents the most frequent site of HCC recurrence, lately reviewed by 66%^2^, leading to the question, whether therapeutic options can be optimized. Currently available diagnostics include radiographic differentiation of indeterminate or hazardous lesions, and alpha-fetoprotein level measurement, which remain limited regarding the analysis of tumor biology^5^.

Molecular profiling could lead to earlier detection of HCC recurrence at a stage, where curative resection and ablation modalities are amenable^5^. Hence, innovative early biomarkers to detect micro metastases are awaited to improve curative therapeutic options in early recurrence after HCC resection.

To optimize diagnosis and monitoring of cancer, non-invasive methods such as liquid biopsies have revolutionized the field of clinical oncology offering ease in tumor sampling and continuous monitoring by repeated sampling^6^. Thereby, tumor-derived extracellular vesicles (EVs) extracted from liquid biopsies are generally present in the blood circulation at early stages of the disease and persist across all disease stages, thus, represent promising potential biomarkers^7–9^. EVs are a heterogenous population of particles released from cells and found in biofluids that consist of lipid-bilayer membrane-coated vesicles^10,11^. EVs cargo consist of miRNAs, RNAs, lipids and proteins^4^. The composition can be altered due to external stimuli such as the blood-ph and hypoxia^4^. Regarding systemic and local tumor dynamics, it was recently shown, that hepatic tumors prepare a comforting microenvironment via release of EVs, initiate tumor angiogenesis and even might evade the immune cell recognition through EV mediated mechanisms^12^.

It has been shown that EVs play a crucial role in cellular communication and metastasis of HCC^10,12^ and could therefore enhance early recurrence diagnosis and thus optimize HCC treatment. As EVs are not only highly heterogeneous in molecular composition but also in their structure of surface proteins, the aims of this study was to characterize the dynamics of EV surface markers in recurrent and non-recurrent HCC patients up to 12 months after surgery. Patients with non-malignant liver disease (NMLD) were used as controls.

## Material and methods

### Patient characteristics and sample collection

The prospective single-center cohort study was conducted at the Johann Wolfgang Goethe University Frankfurt between 2016 and 2019. This study was approved by the ethics committee of the University of Frankfurt (approval number: 321/16). Informed consent forms were signed and obtained from all patients. All experiments were performed in accordance with relevant guidelines and regulations. Blood samples were collected from 36 patients with HCC and 20 patients with non-malignant liver disease (NMLD, control group) before and after surgery. Patients with HCC received segmental resection or hemihepatectomy. After the follow up period of up to 12 months, blood sample and recurrence analysis was taken from 20 HCC patients. Follow up was conducted during routine examination 6–12 months after the surgery. Overall, 16 patients were excluded after 12 months due to loss of follow up and incomplete data set. Out of these 20 patients, 12 (60%) patients were diagnosed with recurrent HCC after a median follow-up period of 12 months.

### Isolation of extracellular vesicles (EVs)

Highly purified EVs were isolated by using EV Isolation Kit Pan Human (Miltenyi Biotec, Bergisch Gladbach, DE)**.** EVs from each patient were analyzed separately. EV isolation was performed by positive selection using MicroBeads recognizing the tetraspanin proteins CD9, CD63, or CD81. The procedure was conducted as follows: 2 ml of Plasma from each sample was pre-cleared by serial centrifugation at 300× g for 10 min, 2000× g for 30 min, 10,000× g for 45 min. Supernatant was collected and transferred into a new tube after each centrifugation step. Next, 50 μL of EV Isolation MicroBeads was added to 2 ml of pre-cleared plasma and incubated for 1 h at room temperature. The labeled EVs are loaded onto a μ Column attached in the μMACS™ Separator. Columns were then washed 4 × with 200 µl isolation buffer and finally the magnetically labeled EVs were eluted by flushing the columns with 100 µl isolation buffer.

### Multiplex surface marker analysis

Serum EV surface expression was analysed by a bead based multiplex EV analysis using MACSPlex Exosome Kit, human (Miltenyi Biotec, Bergisch Gladbach, DE). Detection of 37 EV surface epitopes (CD1c, CD2, CD3, CD4, CD8, CD9, CD11c, CD14, CD19, CD20, CD24, CD25, CD29, CD31, CD40, CD41b, CD42a, CD44, CD45, CD49e, CD56, CD62p, CD63, CD69, CD81, CD86, CD105, CD133.1, CD142, CD146, CD209, CD326, HLA-ABC, HLA-DR DP DQ, MCSP, ROR1 and SSEA-4), including two isotype controls was conducted according to manufacturer’s instructions. Isolated serum EV were stained as follows: 120 µl of each EV sample was incubated with 15 µl of MACSPlex exosomes capture beads overnight at room temperature using a tube rotator on permanent run (12 rpm). Samples were washed with 500 μL MACSPlex Buffer and centrifuged at room temperature at 3000 × g for 5 min. The supernatant was aspirated, 5 μL MACSPlex Exosome Detection Reagent CD9, CD63, and CD81 were added to each sample and incubated for 1 h at room temperature in a tube rotator (12 rpm). Thereafter, 500 μL MACSPlex Buffer was added to each sample and incubated for 15 min in a tube rotator at room temperature. Finally, EV-containing samples were centrifuged at 3000× g for 5 min and EV was collected by aspirating the supernatant, leaving about 150 μL in the tube. Samples were analysed by FACSCanto™ II.

(BD Bioscience). 120 µl isolation buffer was used as background control. The 39 bead populations can be distinguished by different fluorescence intensities detected in the FITC and PE channel.

### Nanoparticle tracking analysis (NTA)

**Figure 1:**
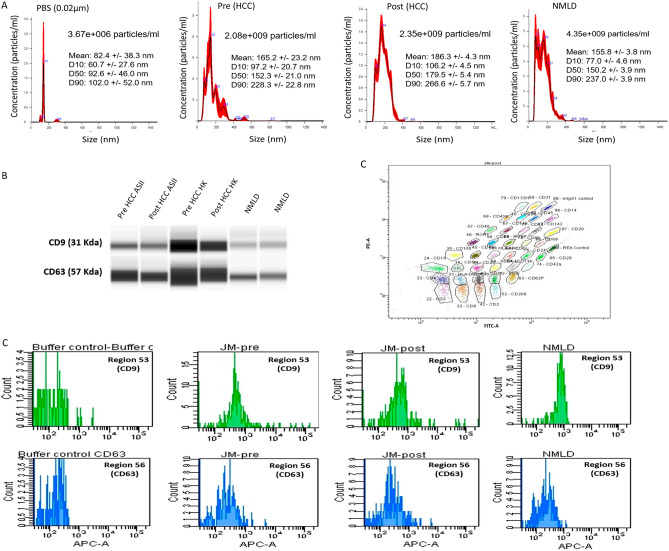
Characterization of EVs: (**A**) NTA analysis showing concentrations and size distribution of Evs isolated from serum of HCC (Pre and Post, n = 5 each)), NMLD patients (n = 5) and resuspension buffer (0.02 µM PBS) used for EVs extraction procedure. (**B**) Example of Protein expression of exosomes markers CD9 and CD63 by Wes protein simple. (**C**) Representative dotplot cluster distribution of 37 EVs markers including 2 controls. (**D**) Representative histograms of EVs stained with CD9 and CD63 detected by MACSPlex EV kit. EVs: Extracellular Vesicles.

Isolated EVs were evaluated by NanoSight NS500 instrument (Malvern Panalytical, Malvern UK) equipped with a red laser (642 nm). The laser illuminated the Nanoparticles and their movement under Brownian motion was recorded for 30 s. The NanoSight particle tracking program (NTA 3.2) was then used to perform nanoparticle tracking analysis (NTA) on six videos. Captures settings were set to the following: camera level of 16, 1300 for the slider shutter, and 512 for the gain slider. The NTA-obtained size distribution profiles were averaged within each sample throughout the six videos, the distribution size profiles were then normalized to total nanoparticle concentrations. Every experiment was run at a 1:100 dilution ((Fig. 1A, Figure S1).

### Western blot analysis of lysed EVs

Wes Simple Western System, a capillary-based electrophoresis (ProteinSimple, Bio-Techne, Minneapolis, USA) was performed according to the manufacturer’s instruction (https://proteinsimple.com). The isolated EVs were directly lysed and identified using primary antibodies (CD9, clone D3H4P and CD63, clone E1W3T), Cell Signaling Technology Europe, Leiden, Netherlands) and (CD81, clone M38), Invitrogen, Thermo Fisher Scientific, Massachusetts, USA). All primary antibodies were diluted at 1:20. Anti-rabbit and anti-mouse detection module served as secondary antibodies. The resulting chemiluminescent signal was detected and analyzed by Compass software for (SW) Simple Western (Fig. 1B, Figure S2).

### Flow cytometry

Measurement of 37 surface proteins was achieved using BD FACSCanto II and analysed by FACSDiva (Becton Dickinson, USA). The median APC fluorescence of each specific bead population obtained from control (buffer only) was subtracted from the signal intensities of the respective beads incubated in the samples. Surface protein expression was measured as fluorescence intensity, the corresponding control antibody was considered as measurement threshold and regarded protein expression below the threshold as negative (Fig. 1C/D, and Figure S3).

### Statistical analysis

Statistical analysis was performed with SPSS (IBM SPSS Statistics, New York, USA) and GraphPad Prism (GraphPad Prism 8.0.0, San Diego, USA) for graph creation. All data showed asymmetric distribution. Statistical analysis included Mann Whitney U test for patient characteristics. Analysis of protein expression was conducted by Friedman test for dependent and Kruskal Wallis test for inter group comparisons plus Bonferroni post hoc test. Results were visualized by median and 95% confidence interval. A level of *p* < 0.05 was considered statistically significant.

## Results

### Patients characteristics

#### HCC and NMLD

The group of HCC patients had a significantly higher age (64.05 ± 10.20 year) than patients with NMLD (48.40 ± 14.13, *p* < 0.0001) and a significantly higher percentage of males (HCC 58.33; NMLD 15%; *p* < 0.0006, Table 1). Percentage of open surgery was comparable within the two groups (HCC 66.67%, NMLD 65%, Table 1).

**Table 1:** Characteristics of HCC, NMLD, R-HCC and NR-HCC patients.

Characteristics	HCC (n = 36)	NMLD (n = 20)	*p* Value HCC versus NMLD	R-HCC (n = 12/20)	NR-HCC (n = 8/20)	*p* Value R-HCC versus NR-HCC
Age (M ± SD)	64.85 ± 11.64	48.40 ± 14.13	< 0.0001	61.88 ± 10.66	67.31 ± 8.48	0.31
Sex (n% males)	58.33%	15.0%	0.0006	75.00%	75.00%	> 0.99
Liver fibrosis	50.0%	–		58.33%	37.50%	0.65
Liver cirrhosis	38.89%	–		25.00%	62.50%	0.17
NASH	22.22%	–		16.67%	12.50%	> 0.99
Type of surgery (n% open surgery)	66.67%	65.0%	0.31	83.33%	75.00%	> 0.99
Tumor volume (cm^3^) (M ± SD)	187.71 ± 247.15	–		183.86 ± 143.97	211.70 ± 243.83	0.85
R Status (% R0)	R0 = 86.11%	–		R0 = 66.67%	R0 = 87.5%	> 0.99
AFP before surgery (ng/ml; M ± SD)	3045.72 ± 11,022.15	2.23 ± 1.05	< 0.0001	3694.49 ± 13,093.02	2163.93 ± 6475.52	0.78
AFP after surgery (ng/ml; M ± SD)	3103.89 ± 10,827.33	2.14 ± 1.10	< 0.0001	4082.81 ± 13,635.75	1888.33 ± 4539.59	0.74

M, Mean; SD, standard deviation; NASH, non alcoholic steatohepatitis; after surgery = within the first week after surgery.

#### HCC: recurrence and non-recurrence

In our study, 60% of patients were diagnosed with recurrent HCC following a median follow-up period of 12 months. When examining the recurrence rates after 1 year, we observed a range of numbers in the literature, spanning from 15%^13^ to 48.1%^14^. Notably, "early recurrence," defined as occurring within 2 years of resection, accounts for over 70% of tumor recurrences^15^. Our findings align with these patterns, which places the recurrence rate in our study within the expected range when compared to other investigations.

The age between patients with recurrence (R-HCC) and non-recurrence (NR-HCC) was comparable (R-HCC 61.88 ± 10.66; NR-HCC 67.31 ± 8.48). 50% of HCC patients had liver fibrosis (R-HCC 58.33%; NR-HCC 37.5%), 38.89% liver cirrhosis (R-HCC 25%; NR-HCC 62.5%) and 22.22% non-alcoholic steatohepatitis (NASH) (R-HCC 16.67%; NR-HCC 12.5%).

The median tumor volume at the time of resection for the HCC group was 187.71 ± 247.15 and showed comparable results between R-HCC (183.86 ± 143.97) and NR-HCC (211.70 ± 243.83) Similarly R Status was comparable between R-HCC (R0 = 66.67%) and NR-HCC (R = 87.5%). The AFP level before and after surgery was similar between the R-HCC and NR-HCC (p = ns, Table 1).

### EV characteristics

Within the representative cohort of 5 patients per group (HCC preoperative/HCC postoperative and NMLD), EVs size distributions were comparable (Fig. 1A, Figure S1) in the NTA analysis. The concentration of particles in HCC preoperatively was : 2.08e + 009 (+ /− 1.27e + 008) particles/ml, and in HCC postoperatively: 2.35e + 009 + /− 7.46e + 007 particles/ml whereas plasma of NMLD group had a higher concentration of : 4.35e + 009 + /− 2.32e + 008 particles/ml. The mean diameter was in HCC preoperatively 165.2 + /− 23.2 nm and for HCC postoperatively 186.3 + /− 4.3 nm. EVs of the NMLD group had the mean diameter of 155.8 + /− 3.8 nm. We confirmed CD9 and CD63 expression as EVs markers in the groups of our study by using Western Blot for analysis of lysed EVs (Fig. 1B, Figure S2).

### EV dynamics in NMLD patients

**Figure 2:**
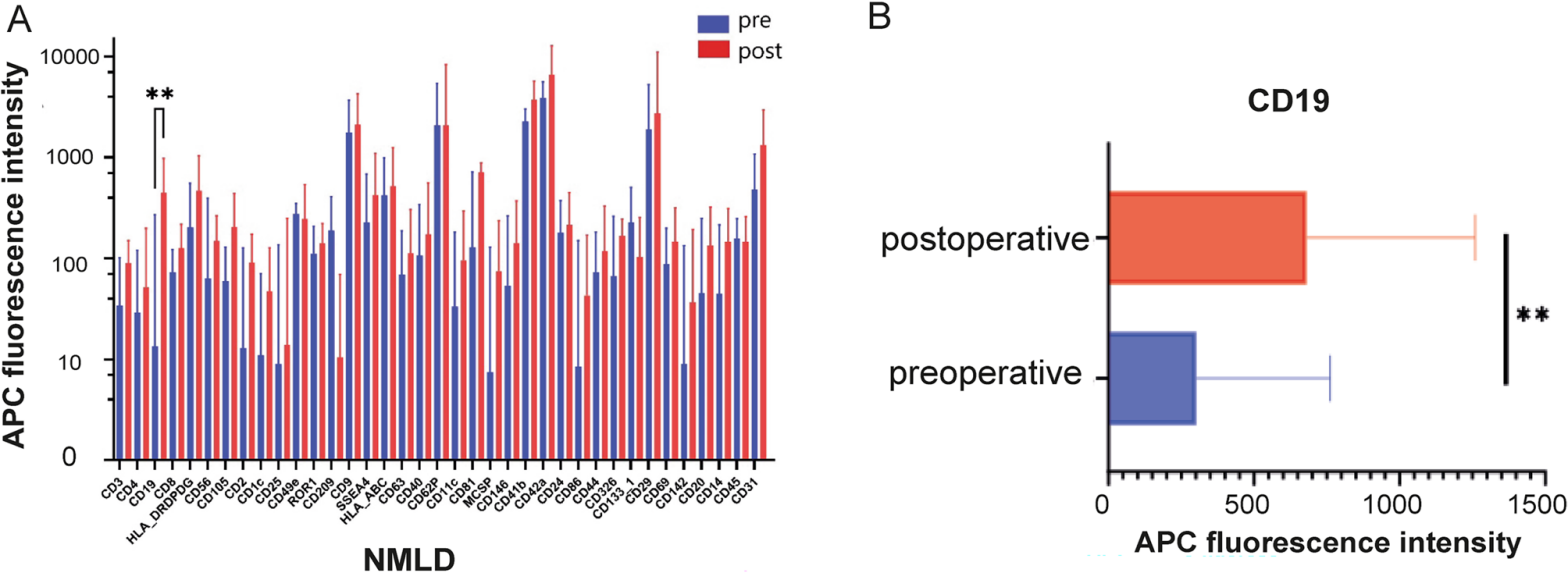
Surface protein expression of EVs in the NMLD group (**A**) for preoperative and postoperative probes in blue and red, respectively. CD19 expression is postoperatively significantly increased in the NMLD group (*p* = 0.0065). (**B**) CD19 expression in EVs of NMLD pre- and postoperatively (**p < 0.01). NMLD: Non-malignant liver disease.

In the NMLD patients, the expression of CD19 was significantly upregulated postoperatively (*p* = 0.0065; Fig. 2). Additionally, the NMLD patients there was a trend of increased protein expressions of MSCP and CD62P postoperatively compared to preoperatively (p = ns).

### EV dynamics in HCC patients

**Figure 3:**
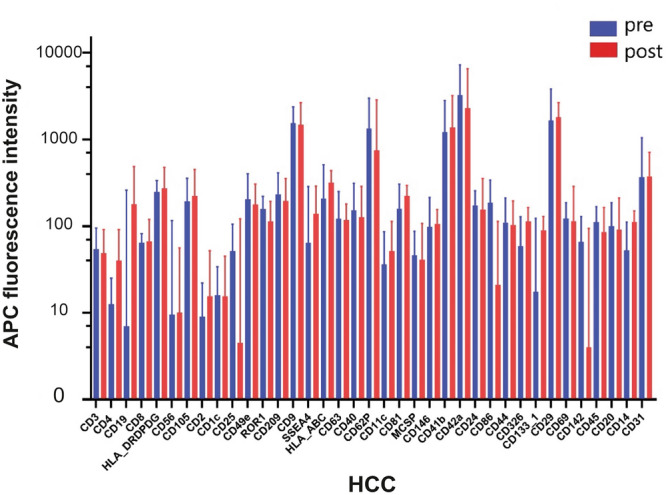
Surface protein expression of EVs in the HCC group (**A**) for preoperative and postoperative probes in blue and red, respectively. HCC, Hepatocellular carcinoma.

**Figure 4:**
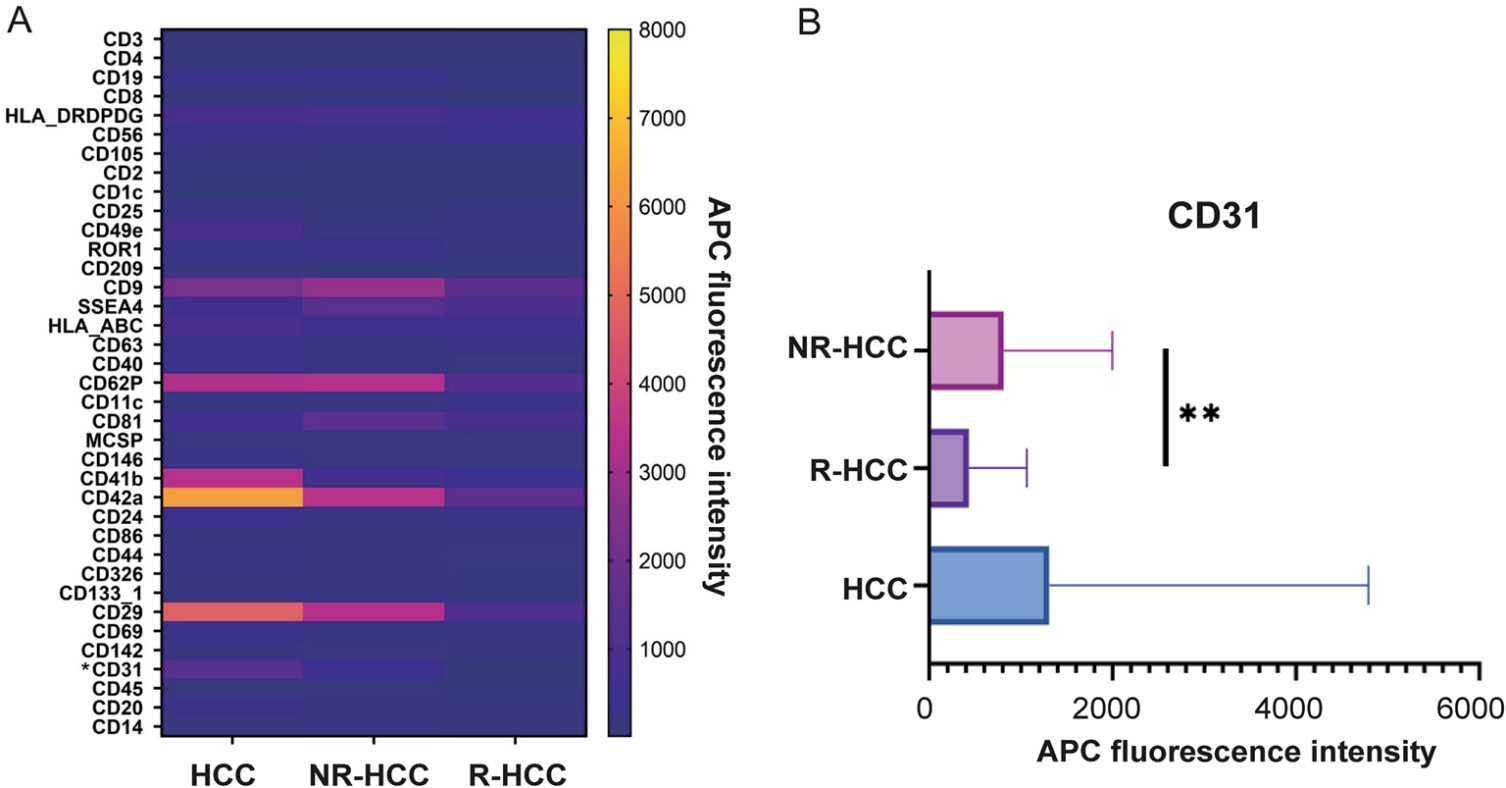
(**A**) Development of EV expression in the R-HCC and the NR-HCC group up to 12 months postoperative. CD31 expression was significantly higher in the NR-HCC group than in the R-HCC group up to 12 months after surgery (*p* = 0.0031). (**B**) CD31 expression in EVs of HCC, NR-HCC and R-HCC (**p < 0.01). HCC, Hepatocellular carcinoma; R-HCC, Recurrence hepatocellular carcinoma. NR-HCC, Non-recurrence hepatocellular carcinoma.

The short-term expression of EV surface proteins in the HCC group remained stable from pre- to postoperative with no significant differences. However, protein expressions of MSCP and CD62P were decreased pre- to postoperative with no significant differences (Fig. 3). Regarding the long-term comparison of the R-HCC and NR-HCC group (n = 20), CD31 expression was significantly higher in the NR-HCC group than in the R-HCC group up to 12 months after surgery (*p* = 0.0031; Fig. 4). Interestingly, the NR-HCC group showed a tendency of higher expressions of CD9, CD62P, CD42a and CD29 up to 12 months postoperativly compared to the R-HCC group, but these differences were not statistically significant (Fig. 4).

### Comparision of EV dynamics between HCC and NMLD patients

**Figure 5:**
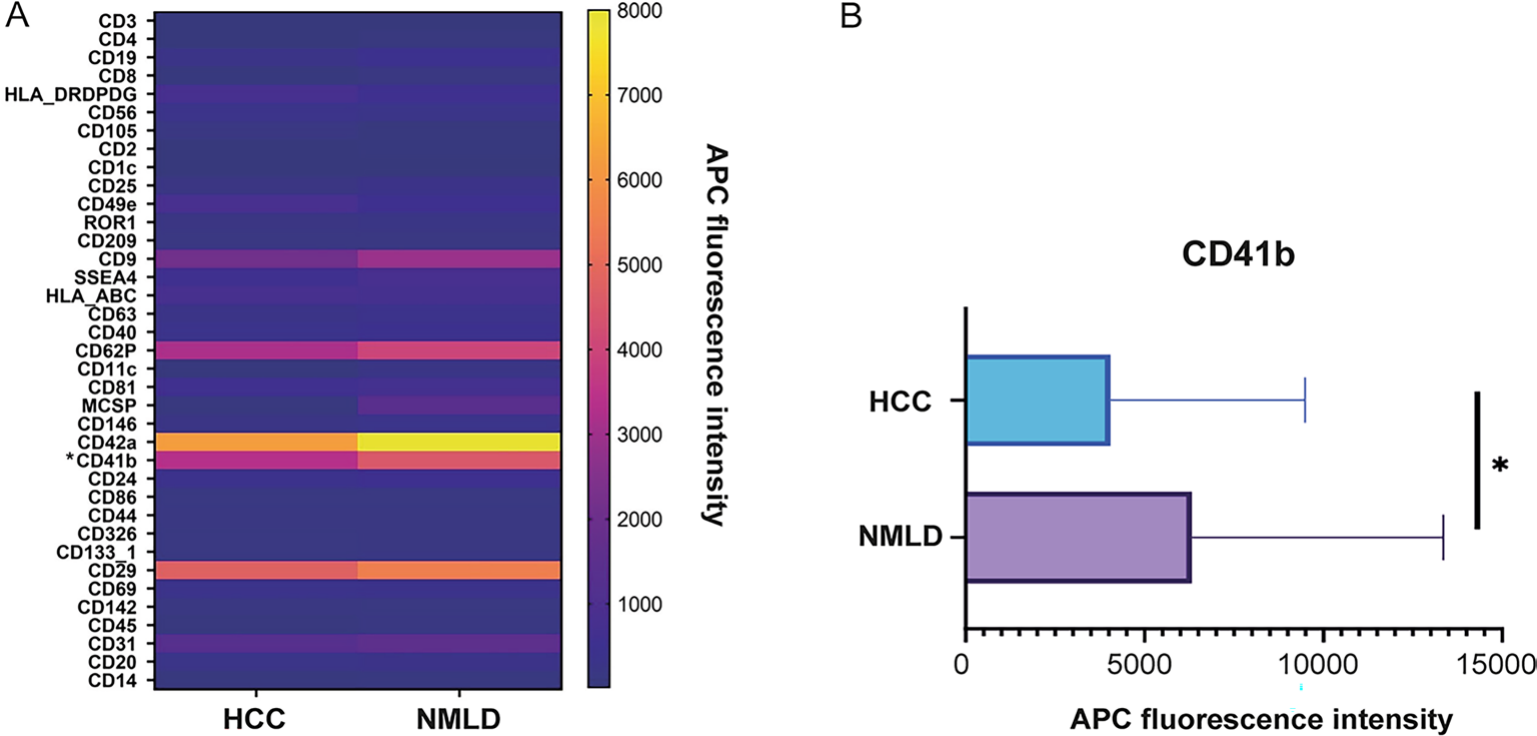
(**A**) Comparison of postoperative EV expression between the HCC and the NMLD group. The HCC group showed a significantly decreased expression of CD41b compared to the NMLD group (*p* = 0.0286). (**B**) CD41b expression in EVS of HCC and NMLD. NMLD: Non-malignant liver disease (*p < 0.05). HCC: Hepatocellular carcinoma.

In this study, a notable difference in the expression of CD41b was observed postoperatively in the HCC patient group compared to the NMLD group, with a statistically significant difference (*p* = 0.0286). However, when considering the aggregate preoperative and postoperative EV expressions, there were no significant disparities between the two patient cohorts. Intriguingly, both HCC and NMLD groups demonstrated a pronounced inclination towards elevated expressions of several markers, namely CD9, CD41b, CD42a, CD29, and CD31 (Fig. 5).

## Discussions

Utilizing liquid biopsy as a promising diagnostic tool for Hepatocellular Carcinomas (HCCs) and predicting their recurrence has been a focus in recent studies^16–18^. While previous research has concentrated on circulating tumor DNAs and microRNAs^19^, our study uniquely aimed to assess extracellular vesicle (EV) surface protein expression as potential novel biomarkers. The analysis of EV surface protein expressions extended up to 12 months post-resection to evaluate their dynamic changes. A significant challenge in HCCs lies in the absence of specific markers for predicting or diagnosing recurrence. This pilot study, for the first time, identified variations in EV surface protein patterns among HCC patients, both in comparison to the Nonalcoholic Fatty Liver Disease (NMLD) group and within HCC patients experiencing recurrence and non-recurrence. The findings contribute novel insights to the quest for effective markers in HCC recurrence prediction and diagnosis.

Recently, surface markers of EVs have gained attention since they indicated presence of HCC and cholangiocarcinoma with increased levels of CD147 and CD133^20^. Additionally, EVs transfer proteins and different types of RNAs, including miRNAs, from highly malignant cells to surrounding cells, promoting HCC migratory and metastatic capacities^21^. This is of particular interest because tumor invasion and migration leads to early and high HCC recurrence rates after resection^22^. Advances in this field may lead to earlier diagnosis and more efficient targeted therapies for HCC. Recent investigations, from Sun et al. (2020) showed promising results regarding HCC specific EV purification^7^.

In the NMLD group, the surgical procedure led to a significant increase in the CD19 rate postoperatively, which is consistent with current literature, as CD19 increment has already been reported in benign hepatic surgery^23^. CD19 is a protein primarily associated with B cells, which are a type of white blood cell involved in the immune response^24^. It is well-known in the context of certain haematological malignancies, particularly B-cell leukemias and lymphomas, and is a target for certain cancer therapies like CAR T-cell therapy^25^. Interestingly, no significant changes in the EVs dynamics were observed due to the surgical resection of the liver in the HCC group. However, decreased granzyme B (+) CD19 (+) B cells were correlated with early recurrence in HCC patients after liver transplantation with poor tumor differentiation, microvascular invasion, increased total tumor diameter, and tumor beyond Milan criteria^26^.

12 months post-surgery, the study unveiled a significant correlation between decreased levels of CD31 and HCC recurrence. CD31, a marker present on both white blood cells and endothelial cells, plays a vital role in angiogenesis—the formation of new blood vessels^27^. This process is crucial for tumor growth and spread and has been linked to promoting HCC metastasis^28–30^. It's noteworthy that CD31 has also been associated with cirrhosis^18^, aligning with our study's results. Based on these findings, we strongly advocate for a more extensive study to reevaluate the significance of CD31 in HCC recurrence.

The surface protein CD41b, similar to a recently discovered platelet-derived exosome marker associated with nonalcoholic fatty liver disease (NAFLD), appears to be elevated in the NMLD group, potentially explaining the substantial increase of CD41b compared to the HCC group in our study. There is existing evidence for CD41-positive EVs in alcoholic liver disease^1^. Despite aligning with other studies, caution should be exercised regarding the findings on CD41b due to reports of its instability in liquid biopsies^1,23^.

One limitation of this conducted study is the small number of patients in the cohort. Furthermore, our analysis focused solely on the surface markers of EVs. Exploring other biomolecules within EVs, such as miRNA, would be valuable for a more in-depth analysis of EV characteristics in correlation with clinical data.

## Conclusions

In conclusion, the present study delved into the comprehensive examination of EV surface patterns in HCC and NMLD. Through our research, we have successfully demonstrated a straightforward and efficient technique for isolating EVs, shedding light on discernible differences in EV surface characteristics between individuals with HCC and those with NMLD. These findings not only contribute to our understanding of the intricate molecular landscape associated with these liver conditions but also provide a crucial foundation for subsequent investigations into EV profiling, especially in the context of HCC recurrence. The insights gained from this study hold the potential to pave the way for the identification of pertinent biomarkers, facilitating the development of more precise and optimized therapeutic strategies in the future.

### Supplementary Information

Supplementary Figure S1.

Supplementary Figure S2.

Supplementary Figure S3.

## Data Availability

The data that support the findings of this study are available from the corresponding author, [MAJ], upon reasonable request.
