# Supplementary figures and images for: Extracellular vesicles as potential biomarkers for diagnosis and recurrence detection of hepatocellular carcinoma
Source: Sci Rep. 2024 Mar 4;14:5322. doi: 10.1038/s41598-024-55888-8 (PMC10912302; doi:10.1038/s41598-024-55888-8)

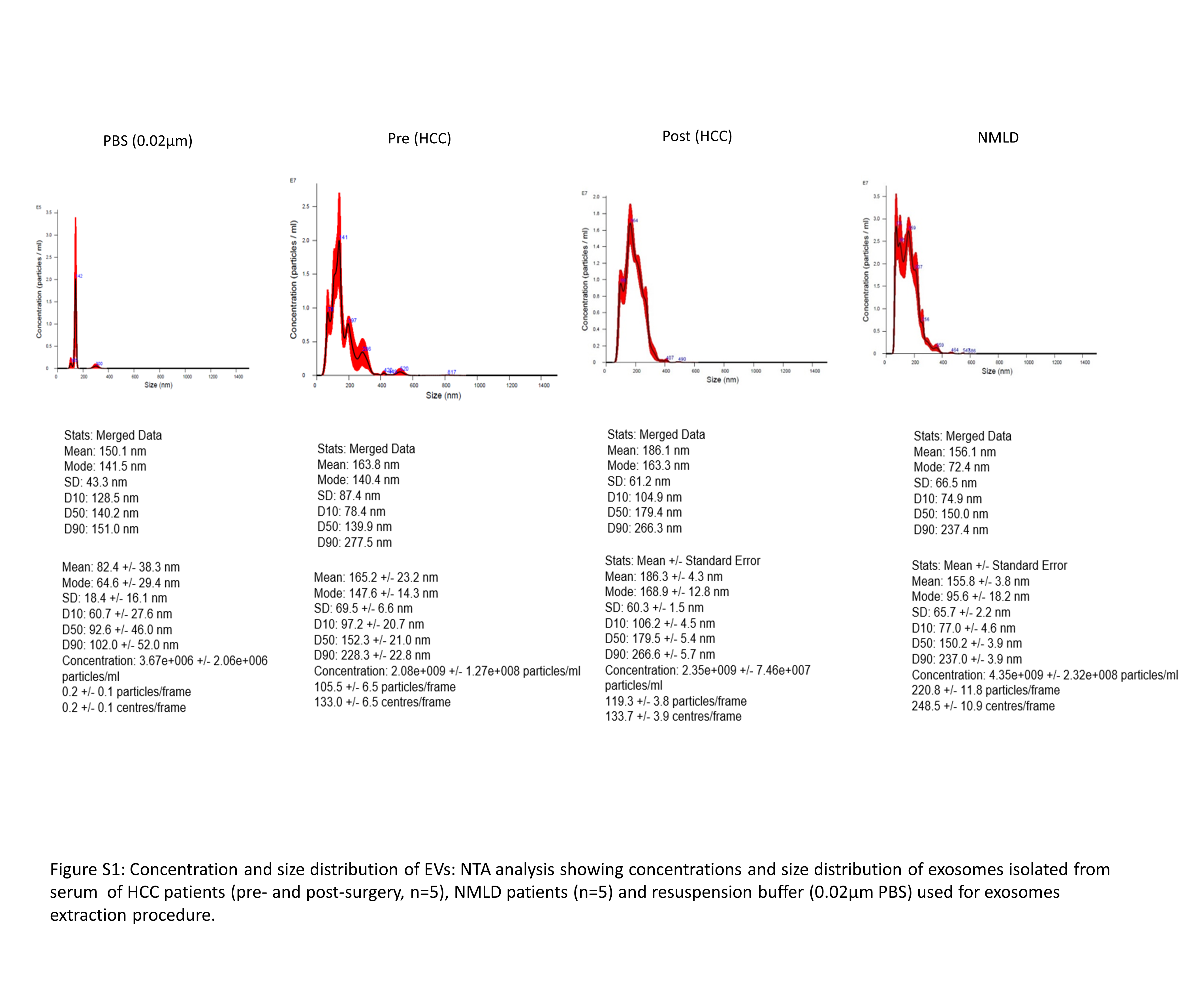

Supplement: Supplementary file 1 — Supplementary Figure S1. [file 41598_2024_55888_MOESM1_ESM.tif]

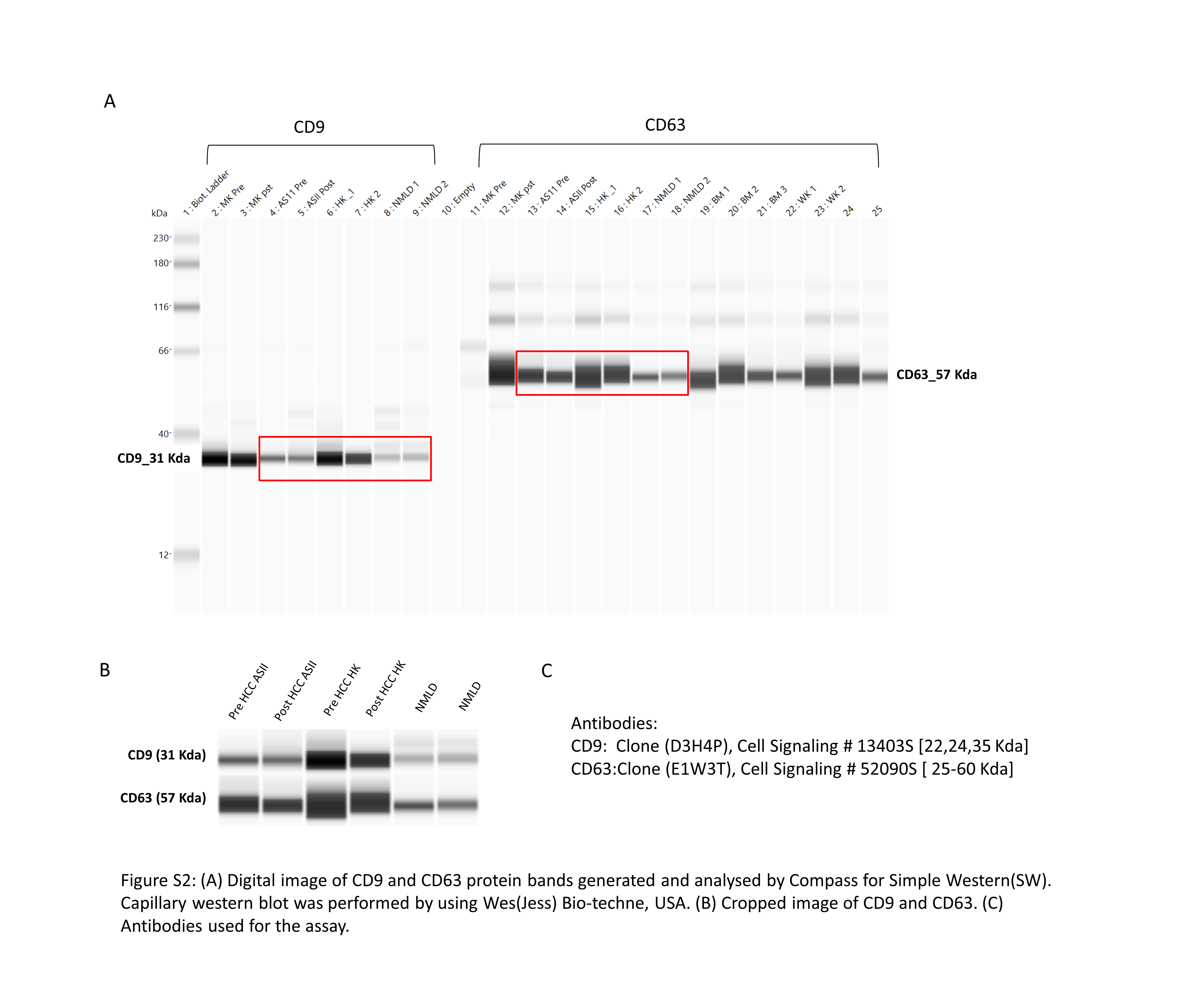

Supplement: Supplementary file 2 — Supplementary Figure S2. [file 41598_2024_55888_MOESM2_ESM.tif]

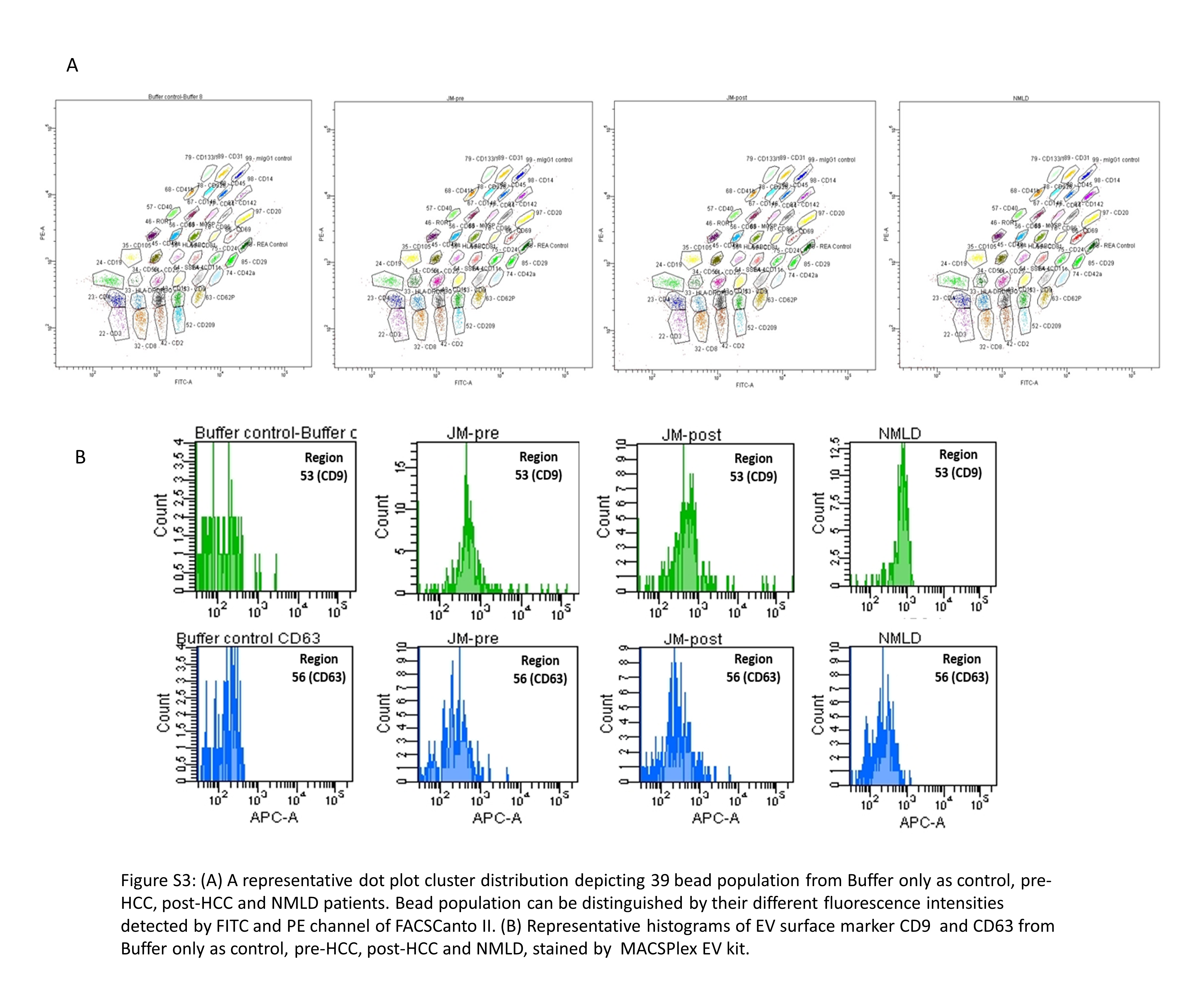

Supplement: Supplementary file 3 — Supplementary Figure S3. [file 41598_2024_55888_MOESM3_ESM.tif]
